# Risk Factors for Sustaining a Second ACL Injury after Primary ACL Reconstruction in Female Football Players: A Study Investigating the Effects of Follow-Up Time and the Statistical Approach

**DOI:** 10.1186/s40798-023-00571-x

**Published:** 2023-05-12

**Authors:** Anne Fältström, Martin Hägglund, Joanna Kvist, Luciana D. Mendonça

**Affiliations:** 1 Unit of Physiotherapy, Department of Health, Medicine and Caring Sciences, Linköping University, 581 83 Linköping, Sweden; 2 Region Jönköping County, Rehabilitation Centre, Ryhov County Hospital, 551 85 Jönköping, Sweden; 3 Stockholm Sports Trauma Research Center, Department of Molecular Medicine & Surgery, Karolinska Institute, Solna, Sweden; 4 Physical Therapy Department, Universidade Federal de Minas Gerais (UFMG), Belo Horizonte, Brazil

**Keywords:** Cox regression, CART, Football, Prediction, Screening

## Abstract

**Background:**

Studies evaluating risk factors for sustaining an anterior cruciate ligament (ACL) injury have different, sometimes contrasting, results. Different follow-up times and statistical approaches may be a reason for these differences. The aim of this study was to explore if different follow-up times and statistical approaches, classification and regression tree (CART) analysis and Cox regression, would impact on the association between various candidate risk factors and ACL injury in female football players. In total, 112 active female football players, 18 ± 8 months after ACL reconstruction (mean age ± SD, 20 ± 2 years), were included and followed for at least 36 months. At baseline, all players underwent assessment of range of motion of knee and ankle joints, functional tests, and answered questionnaires regarding knee function, psychological and personality traits. Nineteen independent variables were included for the CART analysis and for univariable Cox regression and compared using four different follow-up times: 0–12, 0–24, 0–36, and 0–>36 months.

**Results:**

Forty-three (38%) players sustained a second ACL injury. The identified risk factors varied depending on follow-up time both with CART analysis and with Cox regression. CART identified 12 of the 19 independent variables and selected between 5 and 6 of the variables in the four different follow-up times associated with second ACL injury. The accuracy of the different follow-up times for the CART varied between 86 and 93% with 77–96% sensitivity and 70–81% specificity. Cox regression identified two risk factors: knee extension at 0–36 months and 0–>36 months, and time between primary injury and surgery at 0–>36 months. The accuracy varied between 54 and 64% with 44–88% sensitivity and 32–71% specificity.

**Conclusions:**

The identified risk factors associated with a second ACL injury varied depending on the follow-up time and statistical approach used. Thus, in future research on risk factors, the time athletes are followed up and the type of statistical methods used are important to discuss.

**Supplementary Information:**

The online version contains supplementary material available at 10.1186/s40798-023-00571-x.

## Key points

- Identified risk factors for sustaining a second ACL injury varied depending on follow-up time using two different analysis methods: Classification and regression tree (CART) and Cox regression analysis.
- In future research on risk factors, the time athletes are followed up and the type of statistical approaches are important to discuss when interpreting the findings.

## Background

A high proportion of athletes, especially in contact and pivoting sports, sustain not only a first anterior cruciate ligament (ACL) injury, but also a second ACL injury [1–5]. Therefore, a common and important goal in sport medicine is to reduce the risk of both primary and secondary ACL injury. In the clinical setting, screening tests to identify at-risk athletes or risk factors for new ACL injury after primary ACL reconstruction (ACLR) could be performed at various time points after ACLR. In research, screening tests are often performed at a study baseline, i.e. at a specific time point such as at the beginning of the season [6, 7], at the time of ACLR [8], or at the time of return to sport (RTS) [9, 10]. The athletes are then prospectively followed up to register new ACL injuries to analyse the association between baseline tests and risk for future ACL injury. The follow-up times presented in previous research investigating risk factors for ACL injury with different functional performance tests range from 6 months to 8 years [7, 10, 11].

Risk factors to sustain a second ACL injury include young age [4, 9, 12, 13], return to cutting and pivoting sports [4, 8, 14] and RTS before 9 months after ACLR [8, 9]. The importance of other proposed risk factors such as the impact of knee valgus motion in predicting a second ACL injury is debated. Studies with 1–2-year follow-up reported association between excessive knee valgus motion in a drop vertical jump (DVJ) for a primary or second ACL injury [15, 16], but studies with longer follow-up did not [7, 11]. Knee valgus motion pattern could potentially fluctuate over one or several seasons, and the studied cohorts will change over time regarding, e.g. activity level [2], which is an important factor for the risk to sustain a second ACL injury [4, 8, 14]. The problem is that functional performance is dynamic and changes over time, and thus also the association between performance and injury risk. The standard procedure in many studies on risk factors is to include a baseline functional test and then assume that this would be associated with new injury occurrence regardless of how long it has been between the functional performance test and the new injury. Thus, different follow-up times may be a reason for the sometimes contrasting results regarding the association between candidate risk factors and subsequent ACL injury. In addition, the statistical approach used can also contribute to different results related to injury risk [17]. Some studies have explored injury risk adding nonlinear analysis among the factors and make clinical use of these interactions, such as classification and regression tree (CART) analysis [6, 13], but generalized linear approaches such as Cox regression are more commonly used [8–10]. For example, using CART analysis, it was found that hop performance in a triple hop for distance and greater limb asymmetry identified high-risk young female patients for second ACL injury after ACLR and RTS [13]. However, using Cox regression, hop performance was not identified as a risk factor for a second ACL injury in other cohorts [8, 9].

We have previously used CART in a two-year follow-up [6] and reported that an interaction between functional performance, clinical assessment, and psychological factors could identify female football players at high risk for a second ACL injury. In the same cohort, we studied predetermined functional performance test cut-offs and their association with the risk of a second ACL injury where no association was found [18]. Therefore, using the same study cohort, variables and cut-offs, we wanted to study how different statistical methods (CART and Cox regression) and different follow-up periods influence the association between candidate risk factor variables and a second ACL injury occurrence. So, the aim of this study was to explore if different follow-up times and statistical approaches, CART analysis and Cox regression, would impact on the association between various candidate risk factors and ACL injury in female football players. The hypotheses were that: (1) identified risk factors will vary depending on the follow-up time; the association between baseline candidate risk factors and the outcome is likely time-dependent; (2) the association between baseline functional performance tests and psychological readiness for RTS and injury outcome will decrease with longer follow-up times because factors such as muscle strength and hop performance are likely to fluctuate over one or consecutive seasons [19], and psychological readiness to RTS will improve over time [20]; and (3) the association between baseline personality traits and anthropometrics and injury outcome will be more stable regardless of the follow-up time.

## Methods

### Ethics Statement

All players received written and oral information about the study and gave informed consent. The study was approved by the Swedish Ethical Review Authority (Dnr 2012/24–31, 2013/75–32, 2017/324–32, and 2020–01,093) and the Swedish National Knee Ligament Register (SNKLR) board. This research was performed in accordance with the Declaration of Helsinki and in accordance with all relevant guidelines and regulations.

### Participants

Female football players playing at any level, aged 16 to 25 years, with primary unilateral ACLR performed 6 to 36 months earlier were included. Exclusion criteria were having an associated posterior cruciate ligament injury and/or surgically treated injuries to the medial or lateral collateral ligament. For a detailed description of the inclusion procedure, see previous publications [2, 6]. The cohort consisted of 112 active female football players with ACLR (mean ± SD, 18 ± 8 months after ACL reconstruction; mean age 20 ± 2 years; height 168 ± 5 cm; and weight 65 ± 8 kg). This was a sub-cohort of the previously described cohort of 117 players with ACLR in a 2-year follow-up study (Five players dropped out in the long-term follow-up) [2]. In total, 43 (38%) players sustained a second ACL injury (30 ipsilateral and 13 contralateral ruptures).

### Procedure

This is a study using CART analysis and Cox regression with 19 independent variables [6], based on a prospective cohort study analysing risk factors for a second ACL injury. Four different follow-up times from baseline tests were used: 0–12, 0–24, 0–36, and 0–>36 months. At baseline, at the beginning of the football season (January to April), all players completed questionnaires, underwent a clinical assessment, and performed functional performance tests (Table 1). The procedure has been described in detail previously [2, 6, 21]. The independent variables included were the same as those used in previously published data [6]. Nineteen of the baseline variables were included (Table 2).

**Table 1:** Baseline questionnaires, anthropometric measurements, and functional performance tests

Baseline measurements	Description, evaluated
*Questionnaires*	
International knee documentation committee subjective knee Evaluation Form (IKDC) [22, 23]	Knee symptoms, function, and activity limitations in daily living and sports. 0–100, higher score is better
ACL-Quality of Life (ACL-QoL) [24]	Knee function, knee-related pain, symptoms, and quality of life. 0–10, higher score is better
ACL-Return to Sport after Injury (ACL-RSI) [25, 26]	Psychological readiness to return to sport after ACL injury. 0–10, higher score indicate better readiness
Swedish Universities Scales of Personality (SSP) [27]	Thirteen personality traits (somatic anxiety, psychic anxiety, stress susceptibility, lack of assertiveness, impulsiveness, adventure seeking, detachment, social desirability, embitterment, trait irritability, mistrust, verbal trait aggression, and physical trait aggression). 0–100, values > 50 indicate higher levels of the personality traits
Sport Multidimensional Perfectionism Scale (SMPS) [28]	Perfectionism in sports. 1–5, higher scores indicate a high degree of perfectionism
*Anthropometric measurements, range of motion*	
Ankle dorsiflexion in a weight-bearing position using a goniometer	Ankle dorsiflexion, degrees
Knee extension measured in the supine position using a goniometer	Knee extension, degrees; negative values indicate knee hyperextension and positive values extension deficit
*Functional performance tests*	
Single hop for distance [29]	Maximum single hop performance, cm
	Taking off and landing on the same foot, with a controlled, balanced landing
5-jump test [30]	Lower limb explosive power, cm
	Standing on both feet, performed a series of five jumps with alternated left and right foot contacts, and landed on both feet
Drop vertical jump (DVJ) [31–33]	Knee motion in the frontal plane, cm
Tuck jump [34]	Movement asymmetries in a plyometric activity. 0–10, lower score indicate better performance
	The players performed repeated tuck jumps for 10 s. Two standard video cameras, one in the frontal and one in the sagittal plane, 5 m and 3.5 m from the test person, respectively, were used. The tuck jump was analysed from the films by the same person according to a clinician-friendly screening tool [35]
Side hop [29]	Hop performance while developing fatigue, n
	Standing on the test leg and jumping from side to side outside two parallel strips of tape 40 cm apart (with their hands behind their back) performing as many jumps as possible for 30 s. If the foot touched the strips of tape, the hop was not counted. The trials were videotaped to enable analysis of successful jumps

**Table 2:** Descriptive baseline demographics separated into those who did or did not incur a new ACL injury

	Players with ACLR (*n* = 112)		
	Secondary ACL Injury (*n* = 43)	No Secondary ACL Injury (*n* = 69)	*P*
Age, years	20 ± 3	20 ± 2	0.620
Height, cm	166 ± 5	169 ± 5	**0.034**
Body mass index, kg/m^2^	22.3 ± 2.3	22.6 ± 2.3	0.501
Graft: all autografts, n (%)			
Hamstrings	41 (95)	69 (100)	0.145
Patellar tendon	1 (2)		
Quadriceps	1 (2)		
Graft diameter, mm	8.0 ± 0.6	8.0 ± 0.6	0.843
1. Time between injury and ACLR, months	3 (4, 0–11)	4 (5, 0–23)	**0.035**
2. Level of play after primary ACLR, n (%)			0.672
Elite (2 top divisions)	7 (16)	7 (10)	
Third–sixth division	32 (74)	54 (78)	
Lowest division or youth play	4 (9)	8 (12)	
3. IKDC (0–100)	84 ± 11	84 ± 11	0.997
4. ACL-RSI (0–10)	6.8 ± 1.8	6.8 ± 1.8	0.900
5. ACL-QoL (0–10)	7.6 ± 1.5	7.6 ± 1.3	0.967
6. SMPS—personal standards (1–5)	3.3 ± 0.8	3.1 ± 1.0	0.184
7. SSP—somatic anxiety (0–100)	53 ± 8	51 ± 9	0.161
8. SSP—psychic anxiety (0–100)	53 ± 10	51 ± 10	0.201
9. SSP—stress susceptibility (0–100)	54 ± 10	50 ± 10	0.073
10. SSP—impulsiveness (0–100)	51 ± 8	51 ± 10	0.616
11. SSP—adventure seeking (0–100)	53 ± 7	55 ± 9	0.391
12. Ankle dorsiflexion, side difference, degrees	0 ± 2.2	0 ± 1.9	0.607
13. Knee extension ACLR leg, degrees^*a*^	− 7 ± 4	− 4 ± 6	**0.011**
14. LSI—single hop for distance, %	98 ± 9	98 ± 8	0.754
15. LSI—side hop, %	98 ± 18	91 ± 23	0.093
16. 5-jump test, cm	900 ± 111	869 ± 89	0.102
17. Tuck jump total points (0–10)	5 ± 2	5 ± 2	0.201
18. Knee collapse ACLR leg in DVJ, cm	2.8 (6, − 4.4 to 10.7)	2.4 (4, − 7.9 to 9.3)	0.405
19. Knee collapse non-ACLR leg in DVJ, cm	4.4 (5, − 4.1 to 9.4)	3.4 (6, − 10.0 to 10.9)	0.167

Values are expressed as mean ± SD, median (IQR, range), or n (%) of participants. Values in bold type indicate statistically significant between-group differences (*P* < . 05). Numbered variables (1–19) are included in the CART and Cox regression analyses. ACL, anterior cruciate ligament; ACL-QoL, ACL-Quality of Life; ACLR, anterior cruciate ligament reconstruction; ACL-RSI, Anterior Cruciate Ligament-Return to Sport after Injury; DVJ, drop vertical jump; IKDC, International Knee Documentation Committee Subjective Knee Form; LSI, Limb Symmetry Index; SMPS, Sport Multidimensional Perfectionism Scale; SSP, the Swedish Universities Scales of Personality

^a^Negative values indicate knee hyperextension

Anthropometric measurements and functional performance tests were conducted as pre-season tests, supervised by the same experienced test leader (A.F.). The measurements were taken in a standard order as presented in Table 1.

For the single hop for distance and the side hop, a Limb Symmetry Index (LSI) was calculated (ACL − reconstructed limb/uninvolved limb × 100), and used as an independent variable. In the DVJ, knee motion in the frontal plane (medial/valgus or lateral/varus knee displacement) was measured with motion analysis software (Dartfish ProSuite; Dartfish Ltd, Fribourg, Switzerland) from video films (Panasonic HC-V500M). Knee motion during the DVJ was calculated in centimetres as the frontal plane displacement of the knee from the initial ground contact (when the feet just touched the ground, X1) to the end of the deceleration phase (deepest knee flexion position, X2) [31–33]. To simplify the measurement, the greater trochanter, the lateral knee joint line, the head of the fibula, lateral malleolus, patella tendon, and centre of the patella were marked on the players with a marker pen. The displacement was measured between the two vertical lines to the centre of the patella (X1–X2) to represent the amount of knee valgus motion in each leg.

Players were allowed three practice trials and three maximum attempts for the single hop for distance, 5-jump test, and DVJ. However if hop length increased in all three hops in the single hop for distance, additional hops were performed until no further increase occurred. For the tuck jump and side hops, a few test hops were performed to get familiarized with the tests.

### Follow-Up of New ACL Injury

The players answered a web-based question regarding the occurrence of any new knee injury on three occasions annually (pre-season, in-season, and post-season) for the first 2 years after inclusion. If a player reported a new knee injury, she was contacted to inquire if it was a new ACL injury (re-rupture or contralateral rupture), and confirmation of the diagnosis was retrieved from medical records. Detailed descriptions of registered new knee injuries and other injuries over the first 2 years have been published previously for the cohort [2].

Approximately 5 years after baseline, the players again received a follow-up question about occurrence of any new ACL injury. All reported new ACL injuries were confirmed from the SNKLR or medical records.

### Statistical Methods

Statistical analyses were performed with IBM SPSS Statistics for Windows (v 27.0; IBM) and OpenEpi software. Mean ± standard deviations (SD) or median and interquartile range (IQR)/range were calculated to characterize the sample. Between-group comparisons were made with the Student’s *t* test (continuous data), Mann–Whitney *U* test (not normally distributed data), or Fisher's exact test (nominal data).

The hypothesis from our first study [6] was that sustaining a new ACL injury is complex and involves nonlinear interactions among several factors. That was the major reason to use CART. A correlation matrix was performed before running the model to exclude highly correlated variables [6]. CART analysis identifies interactions between independent variables and provides a hierarchical association with new ACL injury. Binary recursive divisions of the initial set of data reveal the variables and their respective cut-off points regarding individuals in each category (with and without new ACL injury), until the sub-groups reach a minimum size or no improvement can be made. For each partition, CART analysis considers all variables to decide which one would be the best to split the parent node in two. The total sample (players with ACLR, *n* = 112; node 0) is considered at the beginning, and the final model represents, hierarchically, the strength of association with the outcome factor (new ACL injury).

The following criteria were used to produce the partitions and, consequently, CART growth: a minimum of eight participants in each node to make a division, a minimum of four participants to generate a node [36], and a Gini index of 0.0001 to maximize the node’s homogeneity. We used fivefold cross-validation, a resampling procedure, to estimate better accuracy and improve the level of fit of the CART model. This approach is used to evaluate machine learning models on a limited data sample [36] and as a measure to decrease overfitting. The classification cost was considered symmetric between categories, and the probability of new ACL injury was established as equal between the groups. A receiver operating characteristic (ROC) curve was created to verify the accuracy of the CART analysis. Finally, relative risks (RRs) were calculated for each terminal node of the CART model to investigate the strength of the associations. One separate CART analysis was conducted for each follow-up at 0–12, 0–24, 0–36, and 0–>36 months.

The Cox proportional hazards model is a method to investigate the association between time to event (new ACL injury) and one or more independent variables. The Cox model can be written as a multiple linear regression of the logarithm of the hazard on the independent variables. One fundamental assumption in the Cox model is that the hazards are proportional, which implies that the hazard ratio is constant across time. The proportional hazards assumption is tested by adding an interaction term of time and each independent variable to the model, where a non-significant result (*P* > 0.05) indicates that the assumption is met. In addition, we plotted the scaled Schoenfeld residuals against time, where the residuals should randomly vary around zero to meet the proportional hazards assumption. We also plotted the Martingale residuals against the continuous covariates to detect nonlinearity.

Univariable Cox regression was used to estimate associations between the 19 independent variables and the occurrence of new ACL injury at the four different follow-up times. The time variable used in the Cox regression was the number of days from baseline to an event or to the end of follow-up. Hazard ratios (HRs) were calculated with 95% confidence intervals (CIs). Significant variables from the Cox regression analyses were dichotomized using cut-offs identified from the CART analysis to calculate sensitivity, specificity, accuracy, and positive (PPV) and negative predictive values (NPV) to predict new ACL injury. The significance level was set at *P* < 0.05.

## Results

Descriptive statistics for demographic characteristics and the 19 independent variables included in the analysis for risk factors for the study sample are presented in Table 2.

Identified risk factors associated with a new ACL injury varied depending on the follow-up time and with the statistical approach used. Here, we present results for CART and Cox regression separately with the different follow-up times.

### CART

**Fig. 1:**
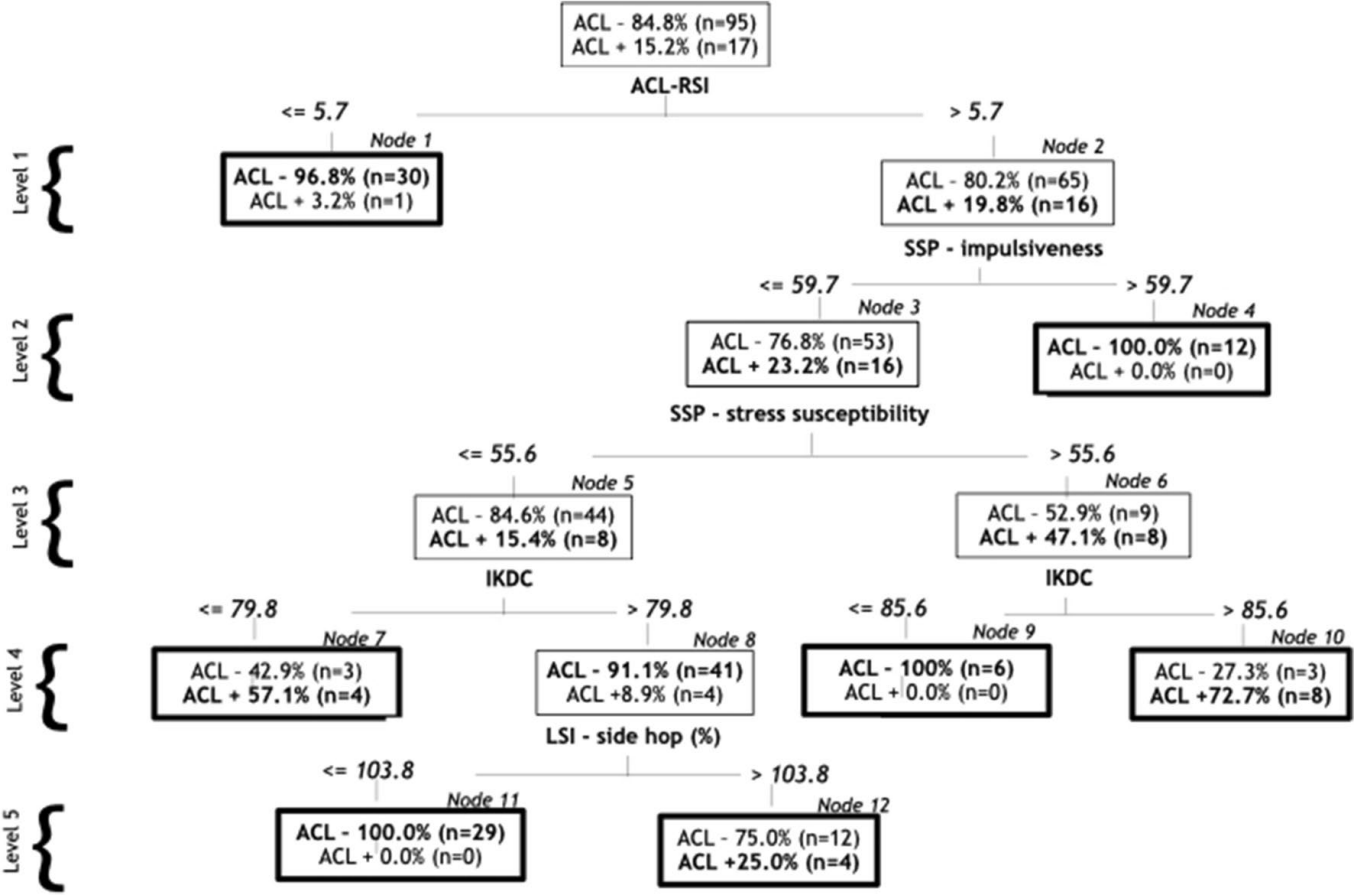
Classification and regression tree (CART) with 0–12-month follow-up for second ACL injury. The bold text in each node (ACL– [no second ACL injury] or ACL + [second ACL injury]) corresponds to the predicted category. All bold boxes indicate terminal nodes. ACL-RSI ranges from 0 (worse) to 10 (best); SSP-impulsiveness, SSP-stress susceptibility, and IKDC range from 0 (lowest) to 100 (highest). ACL-RSI, Anterior Cruciate Ligament-Return to Sport after Injury; IKDC, International Knee Documentation Committee Subjective Knee Form; LSI, Limb Symmetry Index; SSP, the Swedish Universities Scales of Personality

The accuracy of the different CART analyses varied between 86 and 93% with 77–96% sensitivity and 70–81% specificity. CART identified 12 of the 19 independent variables and selected between 5 and 6 of the variables in the four different follow-up times associated with second ACL injury. The first factors selected (level 1, strongest association) by the CART at each follow-up time were as follows: 0–12-month follow-up, ACL-RSI (cut-off 5.7); 0–24-month follow-up, SSP-stress susceptibility (cut-off 44.4); and 0–36-month and 0–>36-month follow-ups, knee extension (cut-off − 4 degrees) (Table 3, Fig. 1, and Additional file 1: Appendix).

**Table 3:** Classification and regression tree analysis with four different follow-up times for players with ACLR (*n* = 112)

	Follow-up times, months			
	0–12	0–24	0–36	0–>36
Secondary ACL injury, n (%)	17 (15)	28 (25)	35 (31)	43 (38)
Sensitivity, %	94	96	77	88
Specificity, %	81	70	80	78
Overall correct classification, %	83	77	80	82
Accuracy, %	93	86	86	87

	Entrance on CART, Level^a^			
	0–12	0–24	0–36	0–>36
**1. Time between injury and ACLR, months**		2		2
2. Level of play, n (%)				
**3. IKDC (0–100)**	4			
**4. ACL-RSI (0–10)**	1			
**5. ACL-QoL (0–10)**			3	3 and 5
6. SMPS—personal standards (1–5)				
**7. SSP—somatic anxiety (0–100)**				4
**8. SSP—psychic anxiety (0–100)**			3	
**9. SSP—stress susceptibility (0–100)**	3	1		
**10. SSP—impulsiveness (0–100)**	2	3 and 4		
11. SSP—adventure seeking (0–100)				
12. Ankle dorsiflexion, side difference, degrees				
**13. Knee extension ACLR leg, degrees**		5	1	1
14. LSI—single hop for distance, %				
**15. LSI—side hop, %**	5		4	3
**16. 5-jump test, cm**		2	2 and 5	2
**17. Tuck jump (0–10)**		3		
18. Knee collapse ACLR leg in DVJ, cm				
19. Knee collapse non-ACLR leg in DVJ, cm				

Significant risk profiles with increased likelihood of sustaining a secondary ACL injury				
Node number and variables included	Relative risk (95% confidence interval)			
	0–12	0–24	0–36	0–>36
**7**: ACL-RSI, SSP-impulsiveness, SSP-stress susceptibility, and IKDC	4.62 (2.04–10.47)			
**10**: ACL-RSI, SSP-impulsiveness, SSP-stress susceptibility, and IKDC	8.16 (3.97–16.78)			
**13**: SSP- stress susceptibility, time between injury and ACLR, SSP-impulsiveness, and knee extension ACLR leg		7.67 (3.15–18.64)		
**4**: Knee extension ACLR leg and 5-jump test			3.39 (2.28–5.05)	
14: Knee extension ACLR leg, 5-jump test, SSP-psychic anxiety, and LSI-side hop			2.70 (1.62–4.50)	
**12**: Knee extension ACLR leg, time between injury and ACLR, LSI-side hop and SSP-somatic anxiety				3.73 (2.25–6.19)
**14**: Knee extension ACLR leg, time between injury and ACLR, LSI-side hop, SSP-somatic anxiety, and ACL-QoL				2.11 (1.31–3.39)

^a^Numbers 1–5 are for the level of entrance in the CART analysis for the selected variables by CART: 1, first level with the strongest association; 2, second level with the second strongest association, etc. Two numbers indicate that the variable is selected on two different levels (e.g. 3 and 4). Variables in bold type were selected by CART. ACL, anterior cruciate ligament; ACL-QoL, ACL-Quality of Life; ACLR, anterior cruciate ligament reconstruction; ACL-RSI, Anterior Cruciate Ligament-Return to Sport after Injury; DVJ, drop vertical jump; IKDC, International Knee Documentation Committee Subjective Knee Form; LSI, Limb Symmetry Index; SMPS, Sport Multidimensional Perfectionism Scale; SSP, the Swedish Universities Scales of Personality

An interpretation of the different follow-up analyses with CART can be given with an example from node 10 with the highest RR (8.16) in the 0–12-month follow-up; players with higher scores in ACL-RSI (cut-off 5.7), lower scores in SSP-impulsiveness (cut-off 59.7), higher scores in SSP-stress susceptibility (cut-off 55.6), and higher scores in IKDC (cut-off 85.6) had an eightfold increased risk of a second ACL injury (Fig. 1). All seven statistically significant profiles were on risk factors for new injury, and no profiles were on protective factors (Table 3 and Additional file 1: Appendix).

### Univariable Cox Regression

Univariable Cox regression did not indicate any significant risk factors in the 0–12- and 0–24-month follow-ups. Univariable Cox regression showed that in the 0–36-month follow-up, only greater knee hyperextension in the ACLR leg was associated with sustaining a secondary ACL injury (HR, 0.935; 95% CI 0.882–0.991; *P* = 0.023, sensitivity 49%, specificity 71%, PPV 44%, NPV 75%, accuracy 64%). In the in 0–>36-month follow-up, greater knee hyperextension in the ACLR leg (HR, 0.936; 95% CI 0.889–0.987; *P* = 0.014, sensitivity 44%, specificity 71%, PPV 49%, NPV 67%, accuracy 61%) and shorter time between primary injury and surgery (HR, 0.898; 95% CI 0.812–0.992; *P* = 0.035 sensitivity 88%, specificity 32%, PPV 45%, NPV 81%, accuracy 54%) were associated with sustaining a secondary ACL injury (Table 4).

**Table 4:** Univariable Cox regression with four different follow-up times for players with ACLR (*n* = 112)

	Follow-up times, months											
	0–12			0–24			0–36			0–>36		
Second ACL injury, n (%)	17 (15)			28 (25)			35 (31)			43 (38)		

	95% CI			95% CI			95% CI			95% CI		
	HR	Lower	Upper	HR	Lower	Upper	HR	Lower	Upper	HR	Lower	Upper
1. Time between injury and ACLR, months	0.916	0.785	1.069	0.893	0.785	1.014	0.903	0.809	1.008	**0.898**	**0.812**	**0.992**
Cut-off < 7.1 months (*n* = 85)										**2.875**	**1.131**	**7.307**
2. Level of play after primary ACLR, n (%)												
Elite (2 top divisions)				1.432	0.239	8.570	1.190	0.320	4.434	1.662	0.487	5.679
Third–sixth division				1.762	0.415	7.473	0.999	0.349	2.863	1.176	0.415	3.331
Lowest division or youth play, reference^*a*^												
3. IKDC (0–100)	1.031	0.980	1.084	1.008	0.974	1.043	1.004	0.974	1.035	1.002	0.976	1.030
4. ACL-RSI (0–10)	1.154	0.878	1.516	1.002	0.818	1.228	1.001	0.834	1.201	1.017	0.862	1.199
5. ACL-QoL (0–10)	1.252	0.845	1.855	1.051	0.794	1.392	1.031	0.802	1.324	1.009	0.805	1.264
6. SMPS—personal standards (1–5)	0.908	0.558	1.478	1.270	0.854	1.888	1.176	0.830	1.666	1.211	0.882	1.661
7. SSP—somatic anxiety (0–100)	1.027	0.973	1.085	1.017	0.974	1.061	1.014	0.977	1.054	1.023	0.989	1.058
8. SSP—psychic anxiety (0–100)	1.013	0.966	1.061	1.013	0.977	1.051	1.015	0.983	1.049	1.018	0.989	1.049
9. SSP—stress susceptibility (0–100)	1.031	0.987	1.078	1.023	0.988	1.059	1.016	0.984	1.048	1.024	0.996	1.053
10. SSP—impulsiveness (0–100)	0.975	0.924	1.029	0.993	0.954	1.033	1.007	0.973	1.043	1.005	0.974	1.036
11. SSP—adventure seeking (0–100)	0.959	0.902	1.020	0.979	0.935	1.026	0.993	0.954	1.034	0.982	0.947	1.019
12. Ankle dorsiflexion, side difference, degrees	1.107	0.869	1.410	1.137	0.945	1.367	0.989	0.840	1.163	0.988	0.908	1.074
13. Knee extension ACLR leg, degrees^*b*^	0.945	0.868	1.028	0.946	0.887	1.010	**0.935**	**0.882**	**0.991**	**0.936**	**0.889**	**0.987**
Cut-off > −4 degrees (*n* = 39)^*b*^							**1.969**	**1.015**	**3.823**	1.756	0.959	3.216
14. LSI—single hop for distance, %	1.024	0.968	1.084	1.014	0.969	1.062	1.011	0.970	1.053	1.008	0.971	1.046
15. LSI—side hop, %	1.014	0.989	1.039	1.014	0.995	1.034	1.008	0.991	1.025	1.013	0.997	1.028
16. 5-jump test, cm	1.002	0.997	1.007	1.003	0.999	1.007	1.003	1.000	1.006	1.003	1.000	1.006
17. Tuck jumps total points (0–10)	0.899	0.695	1.164	0.887	0.725	1.084	0.890	0.744	1.065	0.904	0.769	1.062
18. Knee collapse ACLR leg in DVJ, cm	0.986	0.854	1.139	1.036	0.924	1.161	1.078	0.971	1.196	1.045	0.952	1.147
19. Knee collapse non-ACLR leg in DVJ, cm	1.076	0.928	1.248	1.082	0.965	1.213	1.084	0.979	1.201	1.073	0.979	1.176

Values in bold type are statistically significant (*P* < . 05). ACL, anterior cruciate ligament; ACL-QoL, ACL-Quality of Life; ACLR, anterior cruciate ligament reconstruction; ACL-RSI, Anterior Cruciate Ligament -Return to Sport after Injury; CI, confidence interval; DVJ, drop vertical jump; HR, Hazard Ratio; IKDC, International Knee Documentation Committee Subjective Knee Form; LSI, Limb Symmetry Index; SMPS, Sport Multidimensional Perfectionism Scale; SSP, the Swedish Universities Scales of Personality

^a^No events in the reference group for 12-month follow-up time

^b^Knee hyperextension values are negative and extension deficit values are positive

## Discussion

The main finding was that the identified risk factors for a second ACL injury in female football players with a primary ACLR varied depending on the follow-up time using CART analysis and with Cox regression. Further, the statistical approaches yielded different results with regard to the identification of risk factors for a second ACL injury. In future research on risk factors for ACL injury, the follow-up time of athletes and the statistical approach are important to discuss in relation to the findings.

Our first hypothesis was confirmed regarding the association between risk factors and the follow-up time. In CART, the accuracy (given by the ROC curve) was the highest (93%) in the 0–12-month follow-up. The accuracy dropped to 86% in the 0–24-month follow-up, and then remained stable with longer follow-up (86–87%). One reason for this observed plateau is that prediction models improve with a higher number of injured players, i.e. as with longer follow-up times. We calculated the RR of all terminal nodes for the different CARTs to analyse the strength of each association, not to analyse the models in detail because it was not the purpose of this study. An interesting finding was that higher RRs in CART were found at 0–12- and 0–24-month follow-up (4.62–8.16) and lower RRs were found at 0–36 and 0–>36 months (2.11–3.73). This also suggests that prediction is influenced by the follow-up time.

Cox regression did not indicate any significant risk factors in the 0–12- and 0–24-month follow-ups, but two significant factors (knee extension and time between injury and ACLR) in the 0–36- and 0–>36-month follow-ups. Categories were created based on the cut-offs selected in the CART analyses for the significant values (knee extension, − 4 degrees; time between injury and ACLR, 7.1 months) to analyse the sensitivity, specificity, and accuracy. The sensitivity and specificity varied, but accuracy was consistently low (54–64%). Thus, knee extension and time between injury and ACLR as predisposing factors to ACL injury may be limited. In the current sample, and with the candidate risk factors available, the value of univariable Cox regression to predict who will sustain a new ACL injury is questionable. Previous studies using Cox regression have identified other risk factors to sustain a second ACL injury such as young age at first injury [9, 12], return to cutting and pivoting sports [8, 12], early RTS after primary ACLR [9, 10], and time between injury and surgery [12]. Our cohort already consisted of a high-risk group of young players who had returned to football after ACLR, which could explain the discrepancies. An important aspect to consider is that both CART and Cox analysis are sample-dependent. An example of the sample dependence is the differences from our previously published results with CART analysis with 117 players included [6] instead of 112 as in the present analysis, where the 5-jump test was the first predictor and now the second predictor in the 0–24-month follow-up.

Our second hypothesis that the results from functional performance tests would decrease in importance regarding prediction for longer follow-up times was rejected using CART. The interactions in CART included results from functional performance tests in all different follow-ups. However, this was not the case for psychological readiness to RTS. CART analysis with the 0–12-month follow-up showed that the factor ACL-RSI was selected on the first level, i.e. the strongest association with the outcome, whereas ACL-RSI was not selected with the longer follow-up times. Previous studies have shown an association with ACL-RSI and second ACL injury in follow-up periods of approximately 2 years after ACLR [37, 38], indicating that psychological readiness for RTS may be more important in the short-term follow-up. This confirms the hypothesis that the association between psychological readiness and injury will decrease with longer follow-up times. From the Cox regression analyses, the hypothesis could not be confirmed or rejected because of the non-significant associations of the functional performance tests, psychological readiness, and second ACL injury.

Our third hypothesis that the association between baseline personality traits, anthropometrics, and injury outcome will be more stable regardless of the follow-up time was to some extent confirmed. Personality traits were identified by CART in most of our risk profiles and at different follow-up times. There are some studies regarding personality traits (somatic anxiety, psychic trait anxiety, stress susceptibility, and irritability) and association with injuries in general in football players followed for 3 months [39, 40] and up to 1 football season [41]. Using CART, knee hyperextension was selected at the first level in the 0–36- and 0–>36-month follow-ups. Similarly, using Cox regression, knee hyperextension was also indicated as a risk factor for second ACL injury in the 0–36- and 0–>36-month follow-ups. The association between knee hyperextension and risk of a second ACL injury is not well-studied. However, in a previous study on patients who have undergone ACLR with a patellar tendon autograft, patients with ≥ 6° of hyperextension in the ACLR knee compared with patients with ≤ 3° hyperextension did not show any increased risk for re-ruptures at a mean of 4.1 ± 1.1-year follow-up after ACLR [42]. The evidence is also insufficient regarding generalized joint hypermobility and the risk of graft failure [43]. The importance of personality and anthropometrics in prediction models with longer follow-up need further investigation.

We should be aware that small changes in prediction models could have a great impact on the results. Beischer et al. [9] illuminates the problem in their study; young athletes who returned to sport before 9 months after ACLR had approximately a sevenfold increased rate of sustaining a second ACL injury compared with those who returned at 9 months or later. However, when the authors did further sensitivity analyses and more athletes were included, the rate was lowered to a threefold higher risk. Further analysis with exclusion of the 10% of events with the strongest influence on the analysis showed no relationship between time to RTS and new ACL injury [9]. Another important change in the cohorts depending on time is that players will decrease in activity level and quit football [2]. Return to pivoting sports is probably the most important risk factor to sustain a second ACL injury [14].

Many factors may affect the decision on how long follow-up time is needed in a risk factor study. If the aim is to study, e.g. age, sex, anatomical and surgical factors as potential risk factors, longer follow-up times could be chosen to capture even more secondary ACL injuries, and studies may have as long as 10–20 years of follow-up [44, 45]. Too short follow-up will mean that not all second ACL injuries are captured, even if most secondary ACL injuries occur within the first 2 years after ACLR [12] or the first year after RTS [10].

Traditionally, sports injury prediction has been analysed through linear methods, such as the generalized linear model Cox regression. These methods assume that relationships among explanatory variables and the outcome are linear. However, there has been a paradigm shift regarding sport injury aetiology in recent years, seen as a complex phenomenon, with recommendations to apply statistical modelling that account for complex interactions between explanatory variables [46]. CART provides other benefits rather than just being a nonparametric alternative to Cox. For instance, CART analysis identifies interactions between independent variables, and the method also decreases the risk of overfitting.

Finally, the purpose of this study was to highlight and provide insights for future studies over the importance of addressing follow-up times and statistical methods used in risk factor studies. Psychological and personality factors seem to be important in the short term and greater knee hyperextension in the ACLR leg in the long term. However, the purpose was not to put the findings of the different risk factors into a clinically usable context and to suggest how clinicians should address them in rehabilitation and secondary injury prevention. Previous methodological discussions in risk analysis have been directed towards the variables being examined, time point of the collected variables, the type of variables (continues vs categorical), the amount of data (observations and events), association vs prediction, considerations when modelling the risk of injury, such as the method of data transformation, model validation and performance assessment [47].

The strengths of the present study are the prospective design of a homogeneous cohort of female football players with ACLR, with verification of all new ACL injuries from medical charts. Another strength is the width of factors included in the CART and Cox regression analysis, which made it possible to analyse many different factors regarding prediction and follow-up time.

Some limitations should be acknowledged. This was a relatively small sample with a small number of ACL injuries. Even considering this limitation, we were able to develop accurate models using CART. The fivefold cross-validation approach decreased overfitting for the CART method. Usually, researchers strive to include not only univariable Cox regression, but also multivariable Cox regression. Unfortunately, we could not use traditional multivariable Cox proportional regression analysis with 19 independent variables; the sample was too small, and the model would be overfitting. Another reason was that too few significant factors were identified using univariable Cox regression, despite the risk of false positive values due to multiple univariable analysis, and it would be questionable to perform a multivariable Cox regression. It is common to choose the baseline at the time point of ACLR, but the time for RTS after ACLR vary widely. It is common to choose the baseline at the time point of ACLR, but the time for RTS after ACLR varies widely, e.g. 3–38 months [8, 9]. Therefore, we included players with a range of 6 to 36 months after ACLR. However, using time after ACLR means that the exposure to sport is very different for the included players, and fluctuations in functional performance are expected.

## Conclusions

Identified risk factors associated with a new ACL injury varied depending on the follow-up time and with the statistical approach used. The nonlinear approach (CART) identified more risk factors than the traditional generalized linear approach (Cox regression). Future research on risk factors for ACL (sport) injury should interpret and discuss the influence of follow-up time of athletes and the type of statistical approaches when interpreting the findings.

## Supplementary Information

**Additional file 1:** Supplementary Appendix.

## Data Availability

The datasets used and/or analysed during the current study are available from the corresponding author on reasonable request.

## Abbreviations

ACL: Anterior cruciate ligament
ACLR: Anterior cruciate ligament reconstruction
ACL-RSI: ACL-return to sport after injury
ACL-QoL: ACL-quality of life
CART: Classification and regression tree
CI: Confidence interval
DVJ: Drop vertical jump
HR: Hazard ratio
IKDC: International knee documentation committee subjective knee evaluation form
IQR: Interquartile range
LSI: Limb Symmetry Index
NPV: Negative predictive values
PPV: Positive predictive value
ROC: Receiver operating characteristic
RR: Relative risk
RTS: Return to sport
SD: Standard deviations
SMPS: Sport Multidimensional Perfectionism Scale
SNKLR: The Swedish National Knee Ligament Register
SSP: Swedish Universities Scales of Personality
