# Supplementary material for: Risk Factors for Sustaining a Second ACL Injury after Primary ACL Reconstruction in Female Football Players: A Study Investigating the Effects of Follow-Up Time and the Statistical Approach
Source: Sports Med Open. 2023 May 12;9:29. doi: 10.1186/s40798-023-00571-x (PMC10182191; doi:10.1186/s40798-023-00571-x)
Supplement: Supplementary file 1 — Additional file 1: Supplementary Appendix. [file 40798_2023_571_MOESM1_ESM.docx]

| Supplementary Appendix. |
| --- |

Risk Factors for Sustaining a Second ACL Injury after Primary ACL Reconstruction in Female Football Players: A Study Investigating the Effects of Follow-Up Time and the Statistical Approach.

Sport Medicine Open

Authors: Anne Fältström^1,2^*, Martin Hägglund, Joanna Kvist, Luciana D Mendonça

^1^ Unit of Physiotherapy, Department of Health, Medicine and Caring Sciences, Linköping University, Linköping, 581 83 Sweden.

^2^ Region Jönköping County, Rehabilitation Centre, Ryhov County Hospital, 551 85 Jönköping, Sweden.^*^E-mail: [anne.faltstrom@liu.se](mailto:anne.faltstrom@liu.se); [anne.faltstrom@rjl.se](mailto:anne.faltstrom@rjl.se)

Classification and Regression Tree (CART) analysis for 0-24 (Fig. 2), 0-36 (Fig. 3), and 0->36 months (Fig. 4) follow-up.


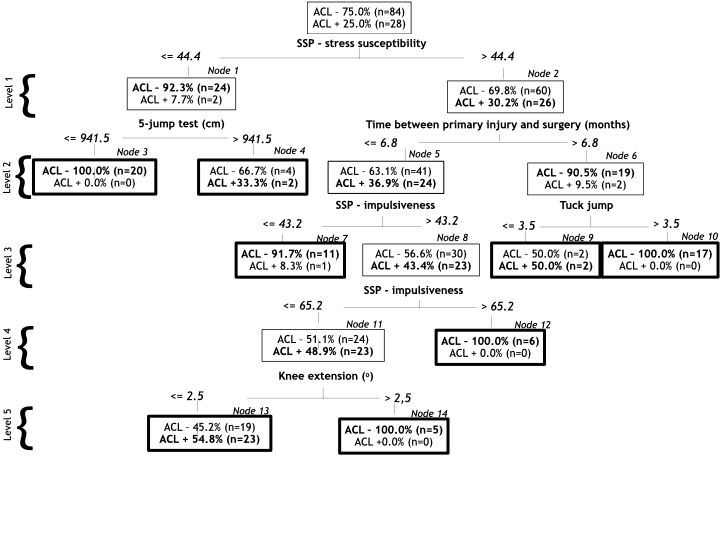


Fig. 2 Classification and Regression Tree (CART) with 0-24 months follow-up for second ACL injury. The bold text in each node (ACL– [no second ACL injury] or ACL+ [second ACL injury]) corresponds to the predicted category. All bold boxes indicate terminal nodes. SSP-impulsiveness and SSP-stress susceptibility ranges from 0 (lowest) to 100 (highest) and Tuck jump 0 (best) to 10 (worse). SSP, the Swedish Universities Scales of Personality.


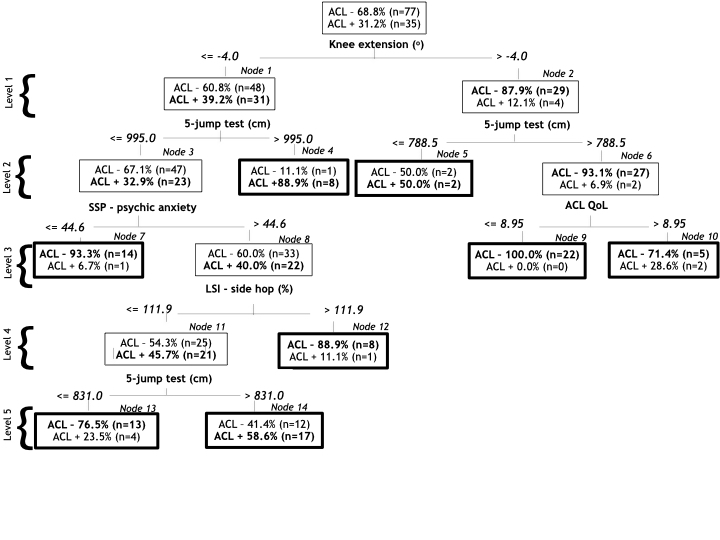


Fig. 3 Classification and Regression Tree (CART) with 0-36 months follow-up for second ACL injury. The bold text in each node (ACL– [no second ACL injury] or ACL+ [second ACL injury]) corresponds to the predicted category. All bold boxes indicate terminal nodes. ACL-QoL ranges from 0 (worse) to 10 (best) and SSP- psychic anxiety from 0 (lowest) to 100 (highest). ACL-QoL, ACL-Quality of Life; LSI, Limb Symmetry Index; SSP, the Swedish Universities Scales of Personality.


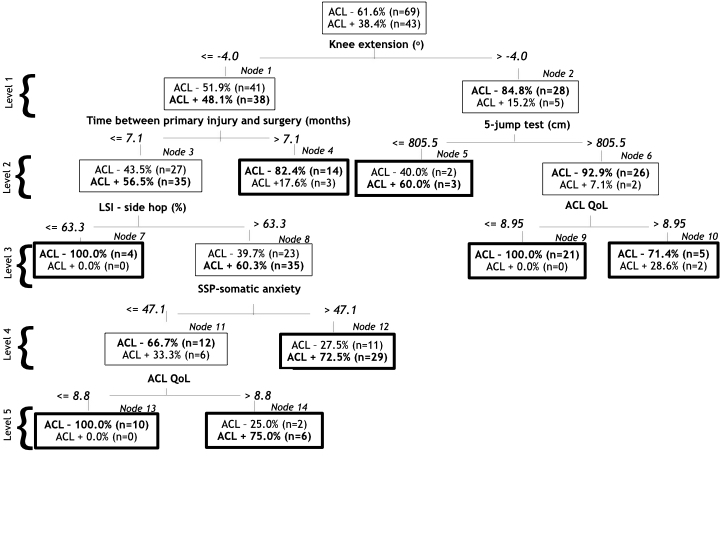


Fig. 4 Classification and Regression Tree (CART) with 0->36 months follow-up for second ACL injury. The bold text in each node (ACL– [no second ACL injury] or ACL+ [second ACL injury]) corresponds to the predicted category. All bold boxes indicate terminal nodes. ACL-QoL ranges from 0 (worse) to 10 (best) and SSP- psychic anxiety from 0 (lowest) to 100 (highest). ACL-QoL, ACL-Quality of Life; LSI, Limb Symmetry Index; SSP, the Swedish Universities Scales of Personality.
